# Estimation of activity concentrations of radionuclides and their hazard indices in coastal plain sand region of Ogun state

**DOI:** 10.1038/s41598-022-06064-3

**Published:** 2022-02-08

**Authors:** O. O. Adewoyin, O. Maxwell, S. A. Akinwumi, T. A. Adagunodo, Z. Embong, M. A. Saeed

**Affiliations:** 1 Department of Physics, Covenant University, Ota, Nigeria; 2 Faculty of Applied Science and Technology (FAST), Universiti Tun Hussein Onn Malaysia (UTHM), Pagoh Campus, Km 1, Jalan Panchor, 84600 Muar, Johor Malaysia; 3 Division of Science and Technology, University of Education Township, Lahore, Pakistan

**Keywords:** Environmental sciences, Natural hazards, Physics

## Abstract

Assessment of natural background radionuclides emanating from the subsurface geological features was carried out with the use of gamma-ray spectrometry at various locations at one of the secondary school in Canaan land, Ota, Ogun State. The activity concentrations of ^226^Ra, ^232^Th and ^40^K were revealed to be 12.66 ± 0.76–42.33 ± 1.37, 44.96 ± 1.41–128.70 ± 1.56, 31.30 ± 1.18–453.85 ± 2.43 Bq kg^−1^ respectively. The mean value of ^232^Th reported higher than the world reference standard of 50 Bq kg^−1^. Moreover, the stations closest to the school’s laboratory were noticed to be prone to more gamma radiations than the other buildings in the school. Similarly, the results of the radiological parameters estimated varied between 86.04–243.7 Bq kg^−1^, 40.02–115.4 nGy h^−1^, 0.049–0.142 mSv y^−1^ and 0.232–0.658 for Ra_eq_, D_(out)_, AEDE and H_ex_, respectively. Although, the results of the radiological parameters did not exceed the world safe limits, higher values of these parameters were reported at some stations closer to the school laboratory. It can be concluded that the school laboratory is prone to more gamma radiation than the class rooms and the administrative block. Therefore, the laboratory instructors and staff, who spend longer time in the laboratory, are more liable to the health risk that could result from years of exposure to gamma radiation in the laboratory.

## Introduction

Humans are continually being exposed to radiation that is emitted from the environment because of the presence of radionuclides^1,2^. This is due to the naturally occurring radioactive materials present in the soil. This can pose as a serious hazard if they are present in high concentrations. This can seriously affect the health of the inhabitants of the community where the radiation is present^2–4^. The concentration of the naturally occurring radionuclides present in the soil can be influenced by man-made activities. Industrial processes such as cement production, coal mining, oil and gas exploration, fertilizer production (phosphate) can enhance the concentration of the radionuclides^1,3^.

Although, there is no place on the earth that is totally free from radioactivity, soil that contains naturally occurring radionuclides that are above the maximum permitted exposure limit can be very dangerous and can seriously affect the health of people living in that environment^5–8^. Therefore, it is important to estimate the amount of radiation people are exposed to from natural sources so as to estimate the associated health risk that is posed to people^6^. Radioactivity is a natural phenomenon. It is part of our everyday life. Natural radioactive materials are present in the air we breathe, and the food we eat; even we ourselves are composed of a certain amount of radioactive materials^9^. Radioactivity also has some useful applications in different areas including agriculture, medicine, mining, geology, archaeology, biology^10,11^ etc.

The intensity of radiation depends on the amount of naturally occurring radioactive materials (NORM) present in the soil and also the time of exposure^12–14^. Possessing the knowledge of the radioactive content in soil is very important in evaluating the radiological hazard it poses to the people within that locality^13,15,16^. Soil with high amount of radionuclides can be a significant source of exposure due to both internal and external radioactivity. Food crops grown in regions where the soil contains high levels of radionuclide may therefore constitute a health hazard^17–20^. It is on this note that this present study was designed to determine the natural radioactive levels (^226^Ra, ^232^Th and ^40^ K) in soil in Faith Academy, Canaan land, Ota, Ogun state and consequently evaluate the radiological hazards associated with it.

## Geology of study area

**Figure 1:**
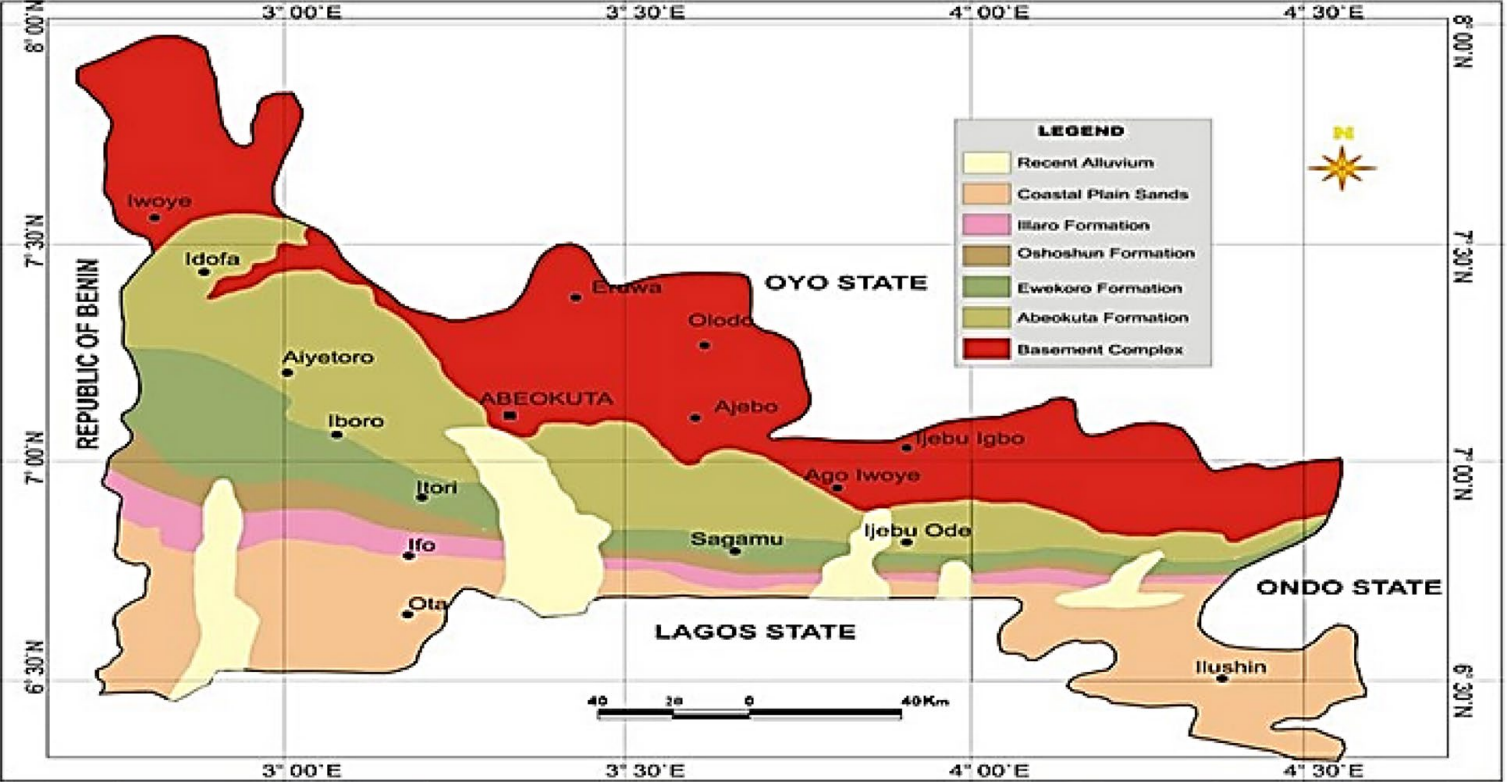
Geological Map of Ogun State, showing the area of study^23^.

The study area lies within Ogun State, which is bounded in the west by Benin Republic, in the south by Lagos State, in the north by Oyo and Osun States, and in the east by Ondo State (Fig. 1). The physiography of the study area is an extensive lowland that is undulating with a gently sloping dissected escarpment known as Southern upland^21^. Further information on the geology of the area of study is captured in^22^.

The area of study is a secondary school premises in Canaan land, Ota in Ogun State, which is located between 6^0^ 40^′^736^″^–6^0^ 40^′^ 692^″^ N and 30 10^′^ 213^″^–30 10^′^ 270^″^ E.

## Materials and method

### Field design

**Figure 2:**
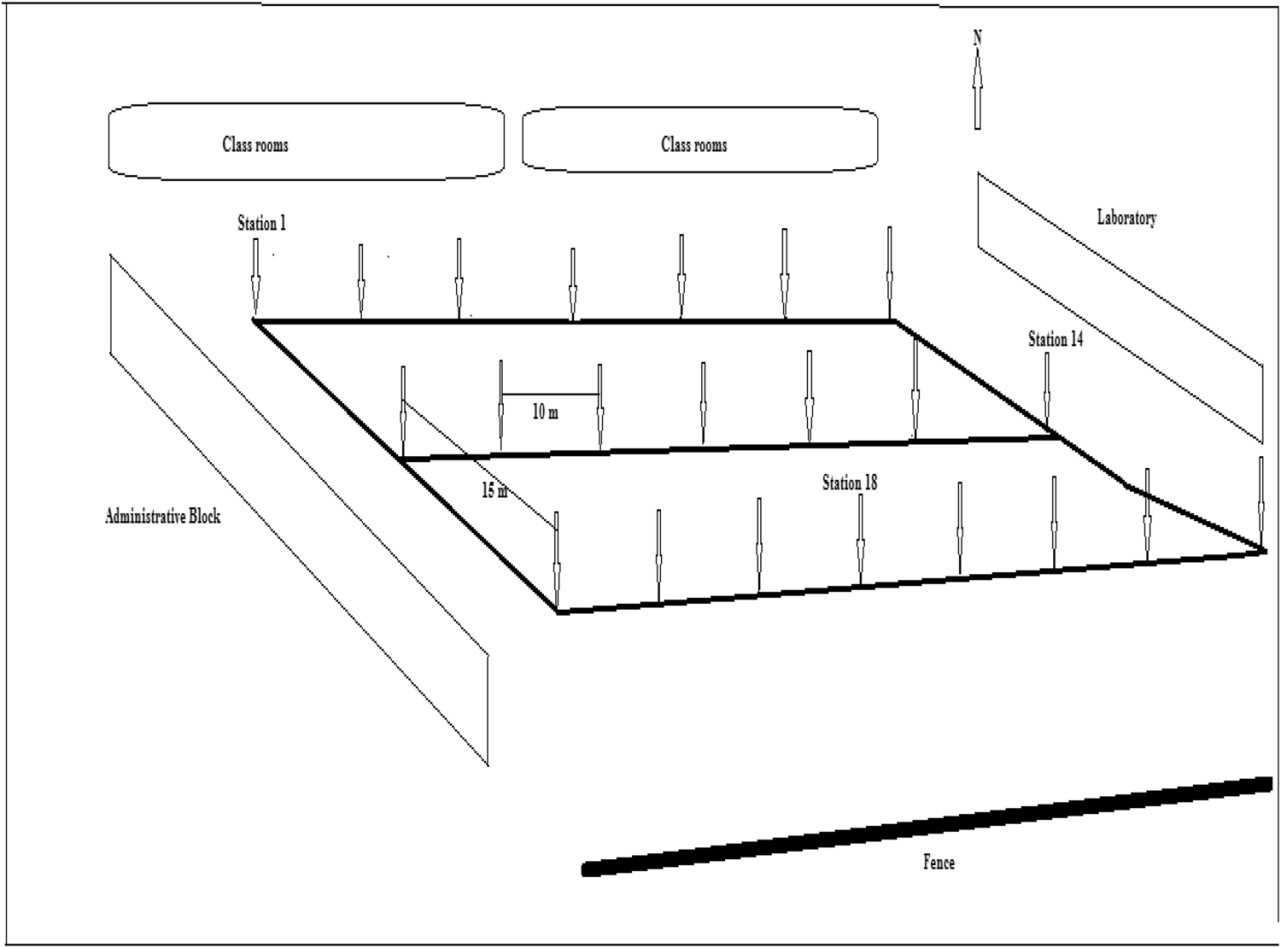
Base map of the Study Area.

The equipment used for this survey is a portable hand held gamma detector with model number RS-125 Super Spec. This is a device that is very prominent for in-situ measurement in radiometric method. This equipment operates on large NaI(Tl) crystal with an energy range from 30 to 3000 keV. It is a single button control device with the ability to auto-stabilize on naturally occurring radio elements. In addition, it is capable of measuring U and Th in ppm and K in % depending on the operation mode that is selected^24^. Furthermore, the radiometric method is a geophysical technique that is used to detect and assess the level of radioactive materials emanating from the subsurface of the earth or present in an area. This equipment detects background radiation that is being emitted from geological features of the earth present in an environment^24–26^. The gamma rays are detected by a spectrometer, which counts the number of interactions with a gamma ray of particular energy. Before the measurement of background radiation began, the field to be surveyed was clearly chosen. The chosen field was rectangular in shape and surrounded by blocks of class rooms. This choice was because any radiation emanating from this source would be going directly into the classrooms. After this, the chosen premises of the school, where the survey was done, was divided into three (3) traverses of 15 m inter-traverse spacing and 10 m spacing between each measurement station as presented in Fig. 2. Two (2) tape rules of 50 m length was used for the measurement. A global positioning system (GPS), with an accuracy of ± 5 m, was used to track the coordinates of the location of each survey. Subsequently, the measurement was done by taking four (4) readings at each station, this is done to reduce the errors that may be in the readings as low as possible. The average of the four readings was found and recorded as the reading for each station^27^.

### Field procedure

Before the commencement of the reading, the hand held measuring device was turned on and allowed to stabilize for about 300 s before measurement proper began. At each measurement station, the portable hand held gamma spectrometer was positioned at 1 m above ground level^26,27^. The measurement for each station was taken at 0.5 m to the north, south, east and west of the station. Each measurement took 120 s and a total of 480 s at each station. After each measurement, the reading was taken at other stations already identified for the survey. The device is calibrated according to the standards provided by IAEA before it was used for this survey^28^. This was done in order to ascertain accuracy and uniformity in the measurement that was done. The device is calibrated to measure Potassium (K) in percent (%), Thorium (Th) in ppm and Uranium (U) in ppm which is displayed as the assay reading after the 120 s. The acquired data in ppm and % for U, Th and K are further converted to the standard units according to^20^ and^29^. These readings were afterwards converted to standard unit of Bq kg^−1^ using the existing conversion factor in literature. That is, for ^226^Ra; 1 ppm = 12.35 Bq kg^−1^; ^232^Th; 1 ppm = 4.06 Bq kg^−1^ and ^40^ K; 1% = 313 Bq kg^−1^ according to^29,30^. The measurement on each traverse was concluded before embarking on the measurements on the other traverses. A number of 7 stations were surveyed on each traverse and 21 stations for all the 3 traverses. However, according to literature^2,30,31^ the formula for estimating the activity concentrations of each radionuclide is presented below as

1 $$A_{s} \left( {Bqkg^{ - 1} } \right) = \frac{{C_{a} }}{{\varepsilon P_{{rM_{s} }} }}$$

where, A_s_ = the activity concentration, C_a_ = the net gamma counting rate in counts per second, $\varepsilon$ = the detector efficiency of the specific gamma-ray, P_r_ = the probability of gamma emission of energy € of the photopeak and M_s_ = the mass of the sample in kilogram.

### Data processing

After the survey was concluded and the readings were taken from the field, the data acquired was recorded in Microsoft Excel in order to allow for easy and further calculations. The average of the four readings per station was calculated and the averages of total readings for each radionuclide were determined. Moreover, other statistical parameters such as the range, mode and rate of data occurrence could be observed at a glance. Also, other radiological parameters were made easy to estimate.

## Estimation of radiological parameters

### Radium equivalent activity (Ra_eq_)

The radium equivalent activity represents a weighted sum of activities of ^226^Ra, ^232^Th and ^40^ K. It is based on the fact that 370 Bq kg^−1^ of ^226^Ra, 259 Bq kg^−1^ of ^232^Th, and 4810 Bq kg^−1^ of ^40^K would produce the same gamma radiation dose rate. The equation for determining the radium equivalent activity is presented in (2), where each component of the equation is explained in^1,26–30,32^

2 $$Ra_{eq} = C_{Ra} + 1.43C_{Th} + 0.077C_{K}$$

### External absorbed dose rate (D_out_)

This refers to the amount of radiation energy absorbed or deposited per unit mass of substance^30^. The absorbed gamma dose rate in air at 1 m above the ground surface for the uniform distribution of the naturally occurring radionuclides (^226^Ra, ^232^Th and ^40^ K) were calculated using

3 $$D\left( {nGyh^{ - 1} } \right) = 0.462C_{Ra} + 0.604C_{Th} + 0.041C_{K}$$

where all the terms in Eq. (3) are explained in^4,32,33^.

### Annual effective dose equivalent (AEDE)

The annual effective dose rate (AEDR) in mSv y^−1^ resulting from the absorbed dose values (*D*) was calculated using the following formula

4 $$AEDE \left( {mSvy^{ - 1} } \right) = D\left( {nGyh^{ - 1} } \right) \times 0.2 \times 0.7 \times 8760 \times 10^{ - 6}$$

According to^34^, 0.2 and 0.7 in Eq. (4) are the external occupancy factor and 0.7 Sv Gy^−1^ is a factor used to convert the absorbed dose rate to effective dose rate. The AEDE is required to assess the health implications of the estimated absorbed dose rates in the area of study.

### External hazard index (Hex)

The external hazard index (*H*ex) can then be defined as:

5 $$H_{ex} = \frac{{C_{Ra} }}{370} + \frac{{C_{Th} }}{259} + \frac{{C_{K} }}{4810}$$

This index must be less than unity (1) for the radiation hazard to be considered insignificant. Each parameter of Eq. (5) is explained as presented in^30–33^.

## Results and discussion

### Measured natural radiation emanating from the subsurface

The varying distribution of the assessed activity concentrations of radionuclides in the studied area is presented in Table 1. The activity concentration was 12.66 ± 0.76–42.30 ± 1.37, 44.96 ± 1.41–128.70 ± 1.56 and 31.30 ± 1.18–453.85 ± 2.43 Bq kg^−1^ for ^226^Ra, ^232^Th and ^40^ K, respectively. Moreover, it was noted that the lowest value of activity concentration for ^226^Ra occurred at station 11, while the highest value was observed at station 22. Similarly, for ^232^Th, the maximum value of activity concentration was noticed at station 13, while the minimum was discovered at station 1. Finally, the activity concentration of ^40^K recorded its highest value at station 13, while the lowest value was encountered at station 2. The variation in the activity concentration of the radionuclides in the study area could be largely a result of the differences in the distribution of the sediments that composed the geology of the area of study^1–3,25–28^. In addition, this could be mostly a result of the varying deposition of clay content, which is typical of the geological formation of the area of study^35^. The regions that reported high activity concentrations in the area of study could be regions with very high clay composition^22–24^. In addition, the high value of ^232^Th and ^40^K observed at stations 12 and 13 could be due to the remains of the deposit of soil materials that is high in phosphate composition, deposited in the area of study during the construction of the school blocks. From Fig. 2, it can be seen that stations 1 and 2, which recorded lowest values of ^232^Th and ^40^K are bounded to the north by class rooms and the administrative block to the west. In addition, the lowest value of ^226^Ra was observed towards the central portion of the area of study. However, the highest values of ^232^Th and ^40^K coincided at station 13, which is bounded to the east by the laboratory, while the highest value of ^226^Ra occurred at station 22, which is bounded by classrooms to the north and a laboratory to the east. Figure 2 reveals that students in the laboratory are more exposed to naturally occurring radioactive materials emanating from the geological features embedded in the subsurface. The result of ^226^Ra and ^232^Th obtained in this study is far higher than the result of^2,18^, where their results for both ^226^Ra and ^232^Th ranged between 2.9 ± 1.00–31.80 ± 6.00 and 1.40 ± 1.00–14.90 ± 4.00 Bq kg^−1^, respectively. The reason for the variation in results may be the impact of the differences in the geological formation of the two areas. However, the results obtained for ^40^K in this research compared with the values obtained for Quartz diorites in^2,28^. This could be the effect of certain geological features that are prominent in the two study areas. The world mean concentrations for ^226^Ra and ^232^Th have been fixed at a mean value of 50 Bq kg^−1^, while it is 500 Bq kg^−1^ for ^40^ K^30,33,36^. The mean concentrations of ^226^Ra and ^40^K in this study are far lower than the international reference standards, while the average value of ^232^Th is higher than the international recommended standard.

**Table 1:** Result of Assessed Activity Concentrations emanating from the subsurface in the Area of Study.

Stations	^226^Ra Bg ${\mathrm{kg}}^{-1}$	^232^Th Bq ${\mathrm{kg}}^{-1}$	^40^K Bq ${\mathrm{kg}}^{-1}$
1	14.51 ± 0.80	44.96 ± 1.41	93.90 ± 2.13
2	21.00 ± 0.97	46.89 ± 1.44	31.30 ± 1.56
3	27.79 ± 1.09	55.83 ± 1.56	86.08 ± 1.77
4	28.41 ± 1.12	55.32 ± 1.57	70.43 ± 1.76
5	23.47 ± 1.02	58.87 ± 1.60	31.30 ± 1.18
6	35.82 ± 1.17	58.36 ± 1.53	54.78 ± 1.56
7	19.74 ± 0.93	86.58 ± 1.17	242.58 ± 2.06
8	21.00 ± 0.99	45.37 ± 1.42	31.30 ± 2.13
9	22.54 ± 1.00	50.85 ± 1.50	39.13 ± 1.44
10	17.60 ± 0.89	53.19 ± 1.56	101.68 ± 2.13
11	12.66 ± 0.76	73.49 ± 1.50	211.23 ± 3.06
12	21.30 ± 0.97	104.65 ± 1.48	406.75 ± 3.18
13	24.70 ± 1.04	128.70 ± 1.56	453.85 ± 2.43
14	23.77 ± 1.02	50.34 ± 1.50	273.88 ± 3.34
15	16.98 ± 0.86	65.98 ± 1.44	399.08 ± 1.44
16	31.18 ± 1.18	51.56 ± 1.53	164.28 ± 2.70
17	24.08 ± 1.05	54.81 ± 1.56	172.15 ± 2.13
18	28.10 ± 1.11	55.93 ± 1.58	148.63 + 1.96
19	28.71 ± 1.13	75.31 ± 1.38	203.40 ± 3.01
20	34.27 ± 1.28	62.73 ± 1.43	140.75 ± 2.50
21	40.76 ± 1.34	81.20 ± 1.71	344.30 ± 2.41
22	42.30 ± 1.37	66.69 ± 1.54	289.53 ± 3.03
Mean	25.49 ± 1.05	64.89 ± 1.50	181.38 ± 2.22

### Radium equivalent (Ra_eq_)

The results of radium equivalent parameter is presented in Table 2. This parameter was calculated with the use of Eq. (2). The variation in the distribution of radium equivalent across the area of study revealed a range between 86.04 and 243.69 Bq kg^−1^ with a mean value of 134.97 Bq kg^−1^. The minimum value of radium equivalent was observed at station 1, while the highest value was seen at station 13. The result obtained in this is similar to the result of^1,2,28–30^, which could be as a result of the similarity in the geological formations of the study areas. However, the values of radium equivalent obtained for Duwi formation in^1^, is far higher than in the present study by a factor of 2.35. This may be as a result of the absence of this kind of geological composition in the area of the present study. In this study, none of the locations surveyed reported values higher than the international recommended safe limit of 370 Bq kg^−1^.

**Table 2:** Estimated results of radiological parameters considered in this study.

Stations	Ra_eq_ Bq $\text{kg}^{-1}$	D_(out)_ nGy $\text{h}^{-1}$	AEDE $\left(\text{mS}\text{{vy}}^{-1}\right)$	H_ex_
1	86.04	40.02	0.049	0.23
2	90.46	41.36	0.051	0.24
3	114.25	52.54	0.064	0.31
4	112.93	51.79	0.064	0.31
5	110.06	50.34	0.062	0.30
6	123.49	56.30	0.069	0.33
7	162.23	76.22	0.094	0.44
8	88.29	40.35	0.050	0.24
9	98.27	44.98	0.055	0.27
10	101.48	47.12	0.058	0.27
11	134.01	63.19	0.078	0.36
12	202.27	95.94	0.118	0.55
13	243.69	115.35	0.142	0.66
14	116.85	55.31	0.068	0.32
15	142.05	68.17	0.084	0.38
16	177.57	54.55	0.067	0.32
17	115.72	54.01	0.066	0.31
18	119.52	55.44	0.068	0.32
19	152.07	70.91	0.087	0.41
20	134.81	62.24	0.076	0.36
21	183.38	86.03	0.106	0.50
22	159.95	74.72	0.092	0.43
Mean	134.97	61.68	0.076	0.36

### External absorbed dose rate, D_out_

This parameter was estimated with the aid of Eq. (3). Table 2 presents the evaluated result of the external absorbed dose rate. The average value of the evaluated results is 61.68 nGy h^−1^. The lowest and the highest values noted for the external absorbed dose rate varied between 40.02 and 115.35 nGy h^−1^ respectively. Moreover, the lowest value of D_out_ was observed at station 1, while the highest value reported in station 13. The locations of the maximum and minimum results coincided with the radium equivalent activity. According to^19,24^, the standard safe limit for the external absorbed dose rate is 57 nGy h^−1^, which is also the worldwide average value. The mean D_out_ in this study is about five times higher than the dose limit for members of the public in planned exposure situations. It is worthy of note that the principal contributor to this radiological parameter is the gamma radiation from the subsurface geological features^26–28,30–32^. The external absorbed dose rate is proportional to the level of the activity concentration of radionuclides in the environment^37^. It was also noted from Table 2 that stations 7, 11–13, 15 and 19–22 reported values higher than the world average safe limit of 57 nGy h^−1^. The effect of the dose rate in this study is observed to be more concentrated from the central stations towards the eastern part of the field and down the southern part of the study area (Fig. 2). This implies that the occupants of the laboratory will be more exposed to gamma radiation emanating from the subsurface.

### Annual effective dose equivalent (AEDE)

The calculated values of the annual effective dose equivalent ranged between 0.049 and 0.142 mSv with an average value of 0.076 mSv. The maximum value reported at station 13, while the lowest value was noted at station 1 (Table 2). The values of the annual effective dose equivalent estimated in this survey were noted to be lower than the world average of 0.48 mSv. Even though, the values of AEDE are not evenly distributed across the area of study, they are still lower than the international recommended safe limit. This shows that the area of study may be considered safe for the students as far as the annual effective dose equivalent is concerned. The results of AEDE in this study is far higher than the results estimated in the investigation by^3,27,38^ by a factor of 2.30. The difference in the results could be attributed to the variation in the geological compositions of the investigated areas.

### External hazard index (H_ex_)

The result of the external hazard index is presented in Table 2. The calculated values of this index were assessed to vary between 0.23 and 0.66. The estimated average value is 0.36. The lowest value of H_ex_ was noted at station 1, while the highest value was observed at station 13. The estimated results of H_ex_ in this study was found to be lower than the world average of 1 in line with UNSCEAR. This result of external hazard index reported in this study is far higher than the result obtained in the study by^3,4,39,40^. However, this study compares with the values of external hazard index in^2,23^, especially in the geological formation that contains the range of Quartz-diorites and Granodiorite.

Table 3 presents the summary of the results obtained in this study together with the international reference standard. It could be seen that the mean values of activity concentrations for ^226^Ra and ^40^K were lower than the world reference standards. However, the mean activity concentration of ^232^Th in this study is much higher than the international reference standard. Similarly, the results of mean values of Ra_eq_, AEDE and Hex were lower than the world recommended limits while the average value of D_out_ is higher than the world standard.

**Table 3:** Summary of Results in this Study.

	Observed Natural Radioactivity			Radiological Parameters			Hex
	^226^Ra (Bq kg^−1^)	^232^Th (Bq kg^−1^)	^40^K (Bq kg^−1^)	Ra_eq_ (Bq kg^−1^)	D_out_ (nGyh^−1^)	AEDE (mSvy^−1^)	
Maximum	42.30 ± 1.37	128.70 ± 1.56	453.85 ± 2.43	243.69	115.35	0.142	0.66
Minimum	12.66 ± 0.76	44.96 ± 1.41	31.30 ± 1.18	86.04	40.02	0.049	0.23
Mean	25.49 ± 1.06	64.89 ± 1.50	181.38 ± 2.22	134.97	61.68	0.076	0.36
World Reference^7^	33	45	420	370	59	0.480	1.00

## Conclusion

In this study, naturally occurring radioactive material was assessed using a hand held gamma spectrometry equipment (RS-125 Super Spec). The measured results revealed the activity concentrations of ^226^Ra, ^232^Th and ^40^K to be in the following range of values; 12.66–42.33, 44.96–128.7, 31.3–453.85 Bq kg^−1^ respectively. The average value of ^232^Th reported higher than the world reference standard of 50 Bq kg^−1^. Furthermore, the stations bounded to the east by the schools laboratory were observed to be prone to more gamma radiations than the other buildings. Similarly, the results of the radiological parameters estimated varied between 86.04–243.69 Bq kg^−1^, 40.02–115.35 nGy h^−1^, 0.049–0.142 mSv y^−1^ and 0.232–0.658 for radium activity equivalent, external absorbed dose rate, annual effective dose equivalent and external hazard index, respectively. Even though, the results of the radiological parameters did not exceed the world safe limits, higher values of these parameters were reported at stations closer to the school laboratory. This is observed from the central to the eastern region and down to the south-eastern portion of the area of study. It can be concluded that the school laboratory is prone to more radiation than the class rooms and the administrative block. Therefore, the laboratory instructors and staff are more liable to the health risk that could result from years of exposure to gamma radiation in the laboratory.
